# Network-Based Inference Framework for Identifying Cancer Genes from Gene Expression Data

**DOI:** 10.1155/2013/401649

**Published:** 2013-09-01

**Authors:** Bo Yang, Junying Zhang, Yaling Yin, Yuanyuan Zhang

**Affiliations:** School of Computer Science and Technology, Xidian University, Xi'an 710071, China

## Abstract

Great efforts have been devoted to alleviate uncertainty of detected cancer genes as accurate identification of oncogenes is of tremendous significance and helps unravel the biological behavior of tumors. In this paper, we present a differential network-based framework to detect biologically meaningful cancer-related genes. Firstly, a gene regulatory network construction algorithm is proposed, in which a boosting regression based on likelihood score and informative prior is employed for improving accuracy of identification. Secondly, with the algorithm, two gene regulatory networks are constructed from case and control samples independently. Thirdly, by subtracting the two networks, a differential-network model is obtained and then used to rank differentially expressed hub genes for identification of cancer biomarkers. Compared with two existing gene-based methods (*t*-test and lasso), the method has a significant improvement in accuracy both on synthetic datasets and two real breast cancer datasets. Furthermore, identified six genes (*TSPYL5, CD55, CCNE2, DCK, BBC3,* and *MUC1*) susceptible to breast cancer were verified through the literature mining, GO analysis, and pathway functional enrichment analysis. Among these oncogenes, *TSPYL5* and *CCNE2* have been already known as prognostic biomarkers in breast cancer, *CD55* has been suspected of playing an important role in breast cancer prognosis from literature evidence, and other three genes are newly discovered breast cancer biomarkers. More generally, the differential-network schema can be extended to other complex diseases for detection of disease associated-genes.

## 1. Introduction

Treating cancer is quite difficult because more and more evidence has revealed that cancer is a kind of complex genetic disease that involves in multiple genes, proteins, pathways, and regulatory interconnections. In order to provide useful information for cancer treatment, several landmark studies [1–3] were performed to uncover oncogenes or biomarkers of cancer development, progression, or recurrence.

Gene-based approaches have emerged in recent years to identify sets of tumor-related genes, such as the “top-down” approach as defined in [4] or “minimal biological input” in 76-gene Rotterdam signature [5]. These methods usually utilize microarray gene expression profiling technique and differential expression analysis to identify the cancer-associated genes whose expression levels change significantly among patients suffering from cancer. Though they have been applied to identification of biomarkers relevant to cancer developing or progressing, the gene-based approaches suffer frequently from uncertainty of tremendous candidate genes, which limits our comprehension to the way that tumor appears and grows.

To recognize complex interaction patterns, pathways, and overrepresented biological processes, gene set enrichment analysis (GSEA) [6] has been exploited repeatedly in the gene-based approaches. The GSEA focus on groups of genes that share common biological functions or signaling pathways defined, respectively, by gene ontology (GO) [7] or KEGG [8], and so forth. Recent works also demonstrated that the detected biomarkers based on GO analysis and pathway information are more reproducible than individual marker genes [9]. Those biomarkers can also improve classification accuracy by 8% compared to the original 70 genes [1].

Increasing evidence suggests that cancer related genes are usually organized as pathways or gene networks which consist of a group of interacting genes at molecular level. Moreover, gene signatures discovered from previous studies often enrich in common cancer-related pathways and similar biological processes. The opinion seems to be advocated and accepted by many researchers that only those which can significantly enrich in tumor-induced signaling pathways or relative biological processes are helpful and valuable for molecular diagnostics [10].

Several network-based methods have been proposed to identify novel oncogenes, subnetworks, or pathways involved in tumor progression. Chuang et al. [11] applied a protein network-based approach to identify biomarkers by extracting subnetworks from protein interaction databases. They also demonstrated that biomarkers detected with the network-based method are more reproducible than individual marker genes selected without network information. Wu et al. [12] integrated different types of networks and known gene-phenotype association information to compute similarity score and predict disease genes. Fröhlich [13] constructed a consensus signature by mapping different gene signatures on a protein interaction network, in which a clustering algorithm was performed based on shortest path distances of different genes in a protein-protein interaction network. In addition, Chen et al. [14] developed a network-constrained support vector machine approach for cancer biomarker identification. The method results in an improved prediction performance with network biomarkers by integrating gene expression data and protein-protein interaction data. 

Differential network analysis plays a key role for elucidating fundamental biological responses as well as discovering important differences between the different biological states [15]. In contrast with conventional gene-based methods, by performing the differential network analysis, more characteristic genes or subnetworks known to be related to disease development are identified. Valcárcel et al. [16] inferred a differential network from males with normal fasting glucose (NFG) and impaired fasting glucose (IFG), in which shrinkage estimates of the partial correlation are executed for network construction, and then the differences were explored by utilizing statistical tests between the two defined groups (NFG and IFG). Gambardella et al. [17] developed a powerful procedure named DINA to identify tissue-specific pathways using a slightly modified information entropy measure. Although it can discover differences across a set of networks, DINA is not able to detect distinct network topologies that have equal density. Iancu et al. [18] revealed gene coexpression patterns and detected modules using a custom differential network analysis procedure including correlation coefficient, clustering, and permutation test. In addition, West et al. [19] presented differential network entropy and demonstrated that gene expression differences between normal and cancer tissue are anticorrelated with local network entropy changes. These findings may have potential implications for identifying novel oncogenes.

In this paper, we present a novel differential-network based inference framework, called network-based statistical analysis method (netSAM) to detect oncogenes. Using differential network modeling and functional enrichment analysis rather than purely the differential expression analysis of a single gene or pathway, netSAM overcomes some limitations of the gene-based methods, such as uncertainty of identification or unfitness for generalization. The applicability and effectiveness of the netSAM algorithm are demonstrated on simulated and real data through numerous experiments. Our results show that the netSAM outperforms two gene-based methods (*t*-test and lasso) in accuracy, precision, and overlap ratio, and so forth. Furthermore, we applied netSAM to identify breast cancer genes from two benchmark datasets (Wang et al. and Van De Vijver et al.) and obtained a cancer-associated gene signature consisting of 6 genes (*TSPYL5, CD55, CCNE2, DCK, BBC3,* and *MUC1*), which have been proven biologically reasonable via GO and pathway analyses. The literature mining reveals that the resulting signature possesses higher prediction capability compared to previous work, and it would be useful in both predicting metastasis of breast cancer and facilitating treatment decision.

Our contributions in this paper are composed of three aspects. First, a novel gene regulatory network construction algorithm is proposed, and its inference ability is demonstrated accurately and efficiently. Second important contribution is a scale-free property-based informative prior score. Third, another important contribution of the proposed method is the differential-network schema for the identification of oncogenes. This framework can be extended easily to other complex diseases.

The remainder of the paper is organized as follows. In Section 2, we provide all details of the netSAM.  Section 3 presents experimental results and analysis. Conclusions and future works can be found in Section 4.

## 2. Materials and Methods

### 2.1. Differential Network-Based Inference Framework

We propose a new differential network-based scheme netSAM to evaluate the relative importance of genes based on the linkage characteristics of the entire network. Firstly, the netSAM explores the transcriptional regulatory mechanism underlying distinct cancer phenotypes by filtering genes that are differentially expressed as well as by inferring differential network from “case” and “control” samples. Secondly, the netSAM selects the top-scoring interacting genes, which appear to construct the cancer-related subnetwork, as candidate genes of cancer susceptibility. In this process, we assume that the higher score a gene has, the more likely it is a cancer-associated gene. Finally, we investigate the functional enrichment of top-ranked genes and evaluate reliability of the biomarkers. The overall work flow for the present study is described as follows.

Compared to the gene-based methods, the advantages or features of the netSAM include (a) identifying oncogenes by constructing the differential network rather than differentially expressed analysis, (b) focusing on the “hub” genes which provide insights into the functional modules or pathways, and (c) uncovering gene regulatory relationships via network inference as well as characteristic of the scale-free network.

**Figure 1:**
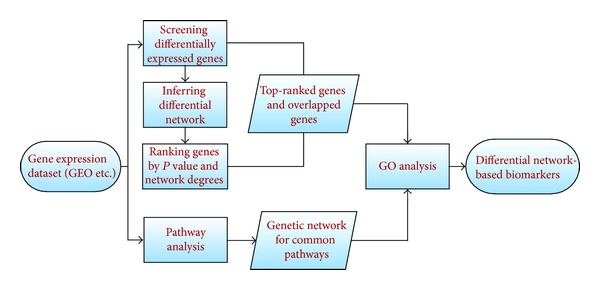
The flowchart of the scheme: the differential network-based identification of cancer biomarkers.

In general, the differential network-based detection of cancer genes includes five steps as described in Figure 1.

**Step 1**

Extracting differentially expressed (DE) genes. In order to remove the features (genes) that show no or minimal discriminatory ability, gene expression data are firstly processed with log2 transformation and then differentially expressed genes are determined by two criteria, fold change of expression level as well as *P* value of Student's *t*-test. In this step, genes whose value of fold-change larger than 2, and meanwhile *P* value less than 0.01 are considered as DE genes.

**Step 2**

Inferring “case” and “control” networks. Based on the reduced features, the individual network is inferred respectively, upon “case” and “control” samples including three steps as the following.

(i)  Computing all regression coefficients *β* that represent interactions between genes, where ${\hat{\beta }}_{ij}=x_{j}^{T}x_{i}\;\;\left(i\neq j,1\leq i,j\leq p\right)$, **x**_*j*_^*T*^ means the transpose of **x**_*j*_ and **x**_*j*_ is the expression profile of gene *j*.
(ii)  Calculating a posteriori score based on likelihood score and informative prior. To make the degree distributions of the underlying network satisfy the scale-free property, the netSAM employs power-law distribution and linear correlation to construct a prior probability distribution. Next, performing boosting updates to obtain an optimal estimation of coefficients *β*.
(iii)  Constructing the gene regulatory network **G** and the adjacency matrix can be formulated as $G_{ij}=\{\begin{array}{cc} 0, & \text{if}\;\;\mathrm{sgn}\left({\hat{\beta }}_{ji}{\hat{\beta }}_{ij}\right)=0\\ 1, & \text{otherwise} \end{array}$, where sgn(·) denotes the sign function.

In the network **G**, the weights of the edges are set to 1 when there is a connection between two genes and 0 otherwise.

Details of Step 2 are given in Algorithm 1 of appendix. Reasons for choosing the boosting regression include (a) the adaptability to achieve the optimal balancing of variance and error, (b) the ability to easily identify genes, and (c) a high computational accuracy and a low calculation time.

**Step 3**

Constructing the differential network. Upon the two networks obtained from “case” and “control” samples, a differential network is established through comparing difference of interactions and subtracting “case” network from “control” one.

Comparison of the “case” and “control” genetic networks will reveal many discrepancies, for example, some interactions are unique and only exist in either networks. As pointed in [15], through network subtraction, the trivial interactions can be removed and detection of differentially represented pathways can be performed. Differentially genetic interactions can also be used to identify novel cancer metastasis-dependent pathways. Thus, network comparison reflects a landscape of differential genetic interactions especially to the genetic disease response.

**Step 4**

Identifying differential network hubs. After building the differential network, the genes are ranked to identify network hubs according to *P* value and degree, that is, number of interactions. With established thresholds for differential interactions (degree ⩾ 5, *P* value ⩽ 0.01), the hubs of the differential network are identified.

The network hubs, that is, genes with many interactions, regulate a variety of cellular functions and are essential for gene-induced lethality or sickness. As previous investigations have shown [17, 20], many hubs in differential interaction network serve as key components of cancer-associated pathways and can be used to discover cancer-induced genes. Our experimental results on breast cancer data also demonstrated that two such differential network hubs, *DCK* and *BBC3*, link to MAPK signaling pathway and metastasis, as reported in [21, 22].

**Step 5**

Prioritizing the network hubs. To identify the most potential cancer genes among the genetic interaction hubs, prioritization of the differential-network hubs is performed.

For the network hubs discovered from the previous Step 4, two ranking methods are performed to select the most promising genes that are associated with cancer. Firstly, GeneRank [23] is employed as computational strategies for gene prioritization in the netSAM. The reason to apply GeneRank is that the algorithm does not require a predefined threshold of important genes and can provide a reordering of genes in terms of their importance and connectivity in the entire network. In GeneRank, a node represents a gene, and an edge is described with expression profile correlation coefficients. Additionally, it requires a connectivity matrix of the network, a vector of differential expression level, and a controlling parameter as its inputs. Specially, to acquire the ranking result based on the connectivity of the network as well as the differential expression level of genes, the controlling parameter is set to 0.5. Secondly, the all hubs are ranked additionally according to their degrees (i.e., number of edges) in the differential network. As the degree can indicate connectivity information and importance of each gene in the whole network, a new ranking result, which is different from that of GeneRank, will be generated by utilizing the sort of degrees. Thirdly, the common genes are selected from the top-ranked hubs of the two previous ranking lists. Thus, a candidate gene-set comprised of essential regulatory hubs of the differential network is obtained. In virtue of the candidate gene-set, a signature consisting of cancer-associated genes is determined finally.

In summary, since the differential network spans the validated differentially expressed genes, the cancer-related genes discovered upon it can provide stronger predictive power than traditional gene-based method. Based on differential network inference framework, the netSAM can be extended easily to a majority of currently known genetic diseases.

### 2.2. Bayesian Criterion and the Posteriori Score

Our method assumes that the individual network **G** can be scored according to its posterior probability given that data is known. The main idea is to choose the edge with the largest score which takes into account the likelihood as well as the prior information of scale-free network. The notion of the most probable network structure is made formal by the Bayesian score criterion, which is simply the posterior probability of **G** given **X**:

(1) $$\begin{array}{c} P\left(G\;|\;X\right)=\frac{P\left(G,X\right)}{P\left(X\right)}\propto P\left(X\;|\;G\right)\cdot P\left(G\right), \end{array}$$

(2) $$\begin{array}{c} \log P\left(G\;|\;X\right)≅\log P\left(X\;|\;G\right)+\log P\left(G\right). \end{array}$$

Here, **X** is matrix of gene expression data, *P*(**X** | *G*) means the (marginal) likelihood probability, and *P*(*G*) means a prior distribution over network structure **G**.

Based on the above discussion, the combined score measure consists of two parts: one is the approximate likelihood, and the other is the network prior information.

Residual sum of squares (RSS) is a measure of the discrepancy between the data and an estimation model. A small RSS indicates a tight fit of the model to the data. Residual sum of squares can be represented as follows:

(3) $$\begin{array}{c} \text{RSS}=\sum_{k=1}^{n}{\left(x_{ki}-{\hat{x}}_{ki}\right)}^{2}={\sum_{k=1}^{n}\left(x_{ki}-\sum_{j=1,j\neq i}^{p}{\hat{\beta }}_{ij}x_{kj}\right)}^{2}, \end{array}$$

where *n* denotes the number of samples and *p* represents the number of features (genes).

Accordingly, we define the approximate log-likelihood score for connectivity strength across genes as follows:

(4) $$\begin{array}{c} \log\left(\text{likelihood}\right)=-\log\left(\text{RSS}\right). \end{array}$$

To capture the mechanisms underlying biology systems and complex networks, a scale-free network prior is applied. Scale-free property means that the frequency distribution *π*(*d*) of the connectivity in a network follows a so-called power law: *π*(*d*) ~ *d* ^−*λ*^, where *d* equals the number of node degrees in the network. The prior information over the network  **G** describing scale-free property can be encoded as the following:

(5) $$\begin{array}{c} \log\left(\text{priori}\right)=\log\left(C^{2}\right),\\ C=r\left(\log\pi \left(d\right),\log\left(d\right)\right),\\ \pi \left(d\right)=d^{-\lambda }\;\left(\lambda >0\right),\;d=\sum_{1\leq m\leq p}\left|{\hat{\beta }}_{mj}\right|. \end{array}$$

To quantify the association or connection between genes, we define the parameter $\hat{\beta }$ as regression coefficient obtained using boosting regression algorithm. Based on likelihood and informative prior, we thus can rewrite (2) as the following:

(6) $$\begin{array}{c} \log\left(\text{posteriori}\;\;\text{score}\right)=-\log\left(\text{RSS}\right)+\log\left(C^{2}\right). \end{array}$$

We applied score function mentioned above to select the fitted edge of network **G** by computing the largest posterior score for all possible gene interaction. Solving this problem leads to the following optimal estimate problem:

(7) $$\begin{array}{c} {\hat{\beta }}_{i}=\underset{\beta_{i}}{\text{argmax}}\;\;\text{posteriori}\;\;\text{score}\left(G;X\right). \end{array}$$

Here, *β*_*i*_ denotes all the coefficient of gene *i*  (1 < *i* < *p*) regressed upon other genes through boosting method.

### 2.3. Functional Enrichment Analysis of Candidate Genes

Gene set enrichment analysis (GSEA) [6] is a computational tool that investigates whether a predefined gene set shows statistical significance. A gene set that contains terms of biological process of gene ontology is constructed, and then overrepresented GO categories are investigated in the detected cancer gene signature by conducting GO analysis using the BiNGO plug-in of Cytoscape [24]. Gene ontology functional enrichment analysis is employed, in which the hypergeometric test is used for functional overrepresentation and false discovery rate for the multiple hypotheses testing correction. Only the corrected *P* values less than 0.05 are considered significant. 

Besides, associations between differential genetic interactions and known pathways are investigated. As shown in the differential network, differential genetic interactions are much more likely to occur among pairs of genes connecting two different subnetworks than among pairs of genes within the same subnetwork. On the basis of these findings, a map of genes and their differential genetic interactions is constructed, in which some of hubs have not been previously linked to cancer development. To validate the newly identified oncogenes, a pathway analysis is performed using DAVID and the parameters are set as default numbers. The significantly enriched functional modules based on KEGG [8] pathway are investigated.

In brief, GO and pathway analyses indicate the effectiveness of the netSAM, which highlights potential application of the method that may be prominent when developing targeted therapeutics. It is also reasonable to believe that the genes detected by the netSAM are highly relevant to cancer either by sharing common cancer-related signaling pathways or by GO functional terms.

## 3. Results and Discussion

In this section, results of experiments with synthetic and real-world data sets are included. We performed a numerical comparison with two existing algorithms (*t*-test [25] and lasso [26, 27]), including GO and pathway analyses. While they provide efficient inference for medium-scale data, *t*-test and lasso typically cannot fully capture the relational complexity for large-scale datasets. Experiments demonstrated the reliability and the effectiveness of the netSAM algorithm. Furthermore, our algorithm occupied a higher position in the accuracy/efficiency trade-off. In addition, validation of the biological reasonability of detected genes as biomarkers was done through analysis of functional enrichment and a vast amount of independent literature.

### 3.1. Simulated Data Experiments

In order to estimate the accuracy of netSAM algorithm and compare its performance with two commonly used gene-based algorithms, that is, *t*-test and lasso, we generated synthetic data sets by using the SynTReN [28], which simulates benchmark microarray datasets with the known underlying biological networks for the purpose of developing and testing new network inference algorithms. Through SynTReN, we simulated a biological network with a known topological structure as well as the corresponding gene expression data. Although numerous tuning parameters can be changed to generate datasets of different sizes and complexity in the software, we kept the default tuning parameters controlling the complexity aspects and only changed the ones controlling noise and the size of dataset being generated.

We generated 100 microarray datasets that consist of 200 genes and 100 sample points (noise *σ* = 0.5); the resulting graphs had approximately 500 connections. For each generated data set, the network structure learned from each method was then compared with the true underlying structure. We ran each experiment 10 times and averaged the results. 

### 3.2. Comparison of the Accuracy and Robustness with *t*-Test and Lasso

Using the synthetic dataset described above, we evaluated the accuracy and robustness of different identification approaches via receiver operating characteristic (ROC), area under curve (AUC), positive predictive value (PPV), and false discovery rate (FDR). ROC, AUC, and PPV will have a value of 1 if the method can perfectly identify the connections in the genetic network. 

**Figure 2:**
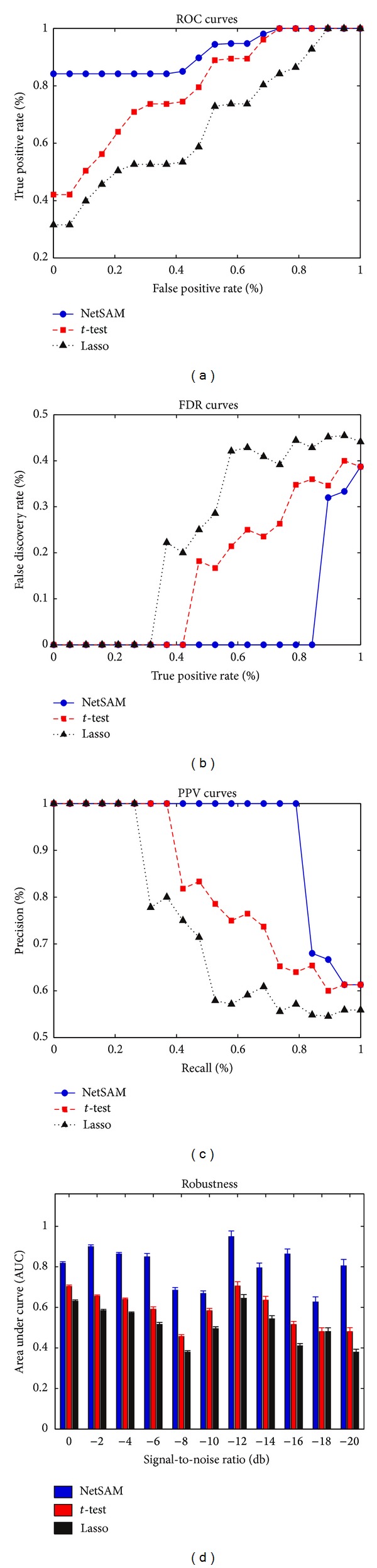
Comparison of accuracy and robustness between netSAM, *t*-test, and lasso on 100 synthetic datasets. (a) ROC curves: true positive rates against false positive rates. (b) FDR curves: error discovery rates against true positive rates. (c) PPV curves: precision versus recall value. (d) Robustness values (AUC versus SNR) are calculated based on five-fold cross-validation, where standard deviations are shown in error bars.

Seen from Figure 2, the netSAM algorithm gets comparatively lower FDR and higher PPV for more edges than *t*-test and lasso. Additionally, robustness, AUC against SNR (signal-to-noise ratio), of biomarker identification over three algorithms is shown in Figure 2(d). In the figure, the average AUC of netSAM is about 0.8, which means that netSAM can select more suited gene biomarkers than *t*-test and lasso. On the contrary, lasso obtains the worst performance over four measures against the other two algorithms. It should be emphasized that these measures depict the inference ability of three algorithms on the same underlying network.

### 3.3. Identification of Breast Cancer-Associated Genes Using NetSAM

In real data experiment, we applied netSAM to breast cancer gene expression microarray dataset previously reported by Wang et al. [29] and Van De Vijver et al. [1]. Only those patients with estrogen receptor positive breast cancer are used as “case” samples, and the remaining estrogen receptor positive samples are assigned to “control” group. Both case and control samples are included in our experiments. After that, the netSAM is applied to the two datasets separately to get two breast cancer gene-set candidates. Finally, they are ranked and intersected for detection of breast cancer genes.

Wang et al. dataset was downloaded from NCBI GEO [30] database GSE2034 [29]. It employs the expression of 22,000 transcripts from total RNA of frozen tumor samples from 286 lymph node-negative primary breast cancer samples that contained 77 estrogen-receptor negative (ER−) and 209 estrogen-receptor positive (ER+) samples, and gene expression profiles were analyzed with Affymetrix Human Genome U133A Array (HG-U133A). Van De Vijver et al. [1] gene expression dataset consists of 295 samples, including 151 lymph node-negative disease and 144 lymph node-positive disease. There are approximately 25,000 human genes which were transcribed and labeled to microarrays for each sample.

Estrogen receptors (ERs) are a group of proteins found inside cells. Once activated, the ER is able to bind to DNA to regulate the activity of different genes. Estrogen receptor positive tumors are the most important subtype of breast cancer. A significant majority (about 70%) of women who died with breast cancer have estrogen receptor positive (ER+) tumors. In these cases, estrogen receptors are overexpressed and referred to as “ER-positive.” While molecular biology has broadened our understanding of breast cancer, we still lack sufficient knowledge of estrogen receptor positive tumors. Aiming at promoting comprehension on estrogen signaling and regulation mechanism contributing to tumorigenesis, we, therefore, focused on patients with estrogen receptor positive breast cancer. In the experiments, we chose 80 samples in Wang et al. and 78 ones in Van De Vijver et al. among the estrogen-receptor positive patients. These selected patients had been diagnosed with metastasis during their follow-up visits within 5 years of surgery and were labeled as “case” group in our study. The remaining 129 and 217 samples, respectively, in the two studies, were then assigned to “control” group.

Using netSAM, 761 and 938 differential genetic interactions were identified totally on the two datasets, respectively, among which 342 and 461 interactions were “positive,” which indicated inducible epistasis, whereas 419 and 477 were “negative,” which indicated suppression. Moreover, we detected 119 hub genes on Wang et al. dataset and 162 on Van De Vijver et al. dataset. A subset of 76 genes was found common between the two candidate gene-sets (119 and 162 genes, resp.). Results of GO and pathway enrichment analyses for the 76 intersection genes are shown in Sections 3.5 and 3.6.

To obtain a breast cancer gene signature, we firstly selected the top 10 ranked genes, respectively, from the two candidate gene-sets (119 and 162). Then, an intersection set was generated between two top 10 ranked gene sets. Finally, six intersection genes were regarded as the breast cancer susceptibility genes, that is, the signature consisting of *TSPYL5, CD55, CCNE2, DCK, BBC3,* and *MUC1*.

**Figure 3:**
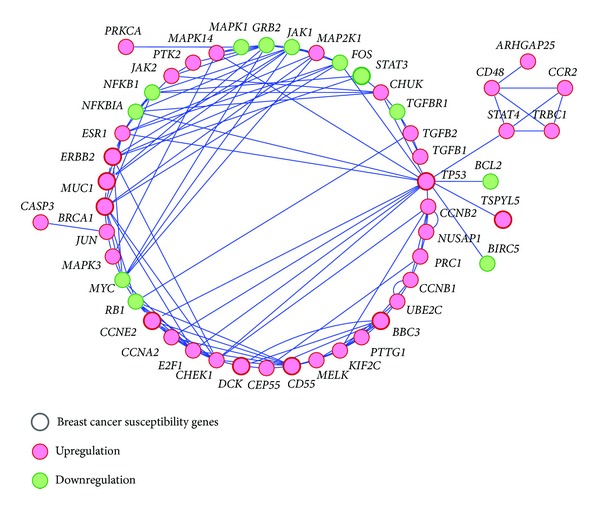
The breast cancer-related genetic subnetwork consisting of the top 50 ranked genes identified through netSAM method from Wang et al. breast cancer dataset. Genes are represented as circles, a significant coregulation between two genes as a line.

In addition, the top 50 ranked genes identified by netSAM from Wang et al. dataset are shown in Figure 3. Seen from Figure 3, not only the known breast cancer metastasis genes (*BRCA1, TP53,* and *ERBB2*) but also the novel cancer susceptibility genes such as *TSPYL5, CD55, CCNE2, DCK, BBC3,* and *MUC1* were identified. These recognized genes interact with many other genes to coregulate the progression and evolvement of breast cancer. The node size is relevant to the breast cancer susceptibility which represents the possibility of gene relating to cancer. Figure 3 was created using Cytoscape [24].

### 3.4. Overlap Analysis between Identified Signature and Literature Reference Gene Set

In this section, we compared the netSAM with gene-based approaches (*t*-test and lasso) on the breast cancer datasets to further examine which method would obtain better signature. To compare overlapped genes through literature mining, we also compiled a list of cancer-associated genes, BCGS (breast cancer literature reference gene set), by collecting genes known to be associated with breast cancer from literature curation and web-based resources. BCGS includes 452 representative cancer-associated genes. The gene symbols were searched as well as extracted from the 1098 PubMed literatures using keyword (breast cancer* gene AND Humans [mesh] OR “Breast Neoplasm” [mesh] AND “Neoplasm Metastasis” [mesh] biological process [go]) in PubMed [31]. These genes form the basis of our “cancer-associated genes” dataset. We then utilized overlap ratio between literature-published gene set BCGS and our candidate genes as evidence of feasibility and the effectiveness of the netSAM.

When two distinct sets share at least one element in common, they are “intersecting” or “overlapping.” In the genomic scenario, we utilized an overlap measure to examine the overlapping capability between the curated gene set BCGS and the cancer gene set identified with different detection algorithms. Specifically speaking, the overlap ratio is defined as the number of intersection genes divided by the number of identified genes.

**Figure 4:**
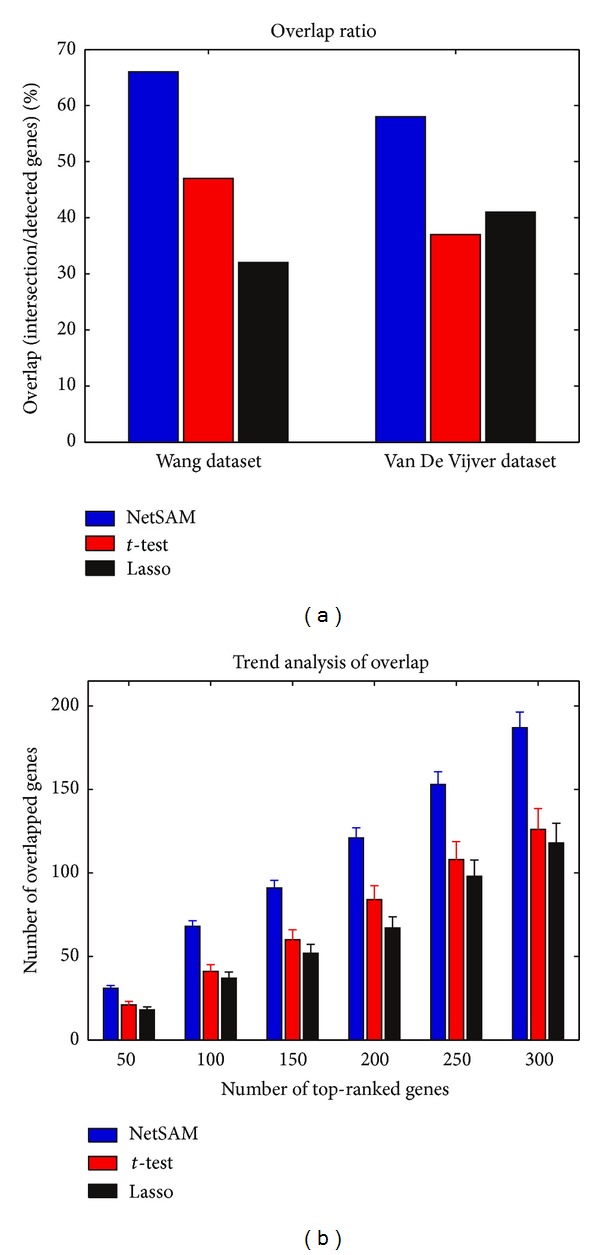
(a) Overlaps of the identified genes using netSAM, *t*-test, and lasso based on Wang et al. and Van De Vijver et al. breast cancer datasets. (b) Trend of overlap: number of overlapped genes versus top-ranked genes (error bars denote standard deviation estimated over 100 tests).

To validate predictive power of netSAM, the overlap ratio and trend analysis of overlap are performed. The comparison results among netSAM, *t*-test, and lasso are displayed in Figure 4 based on Wang et al. and Van De Vijver et al. breast cancer datasets. The comparison of overlap ratio indicates that netSAM can identify some novel cancer-causing genes that are not found by *t*-test and lasso. Only a few of known breast cancer genes were identified correctly by *t*-test and lasso. Seen from Figure 4, the netSAM can identify more overlapped genes than those of the other two methods, which indicates that the netSAM obtains a better reproducibility across different data sets in terms of biomarker identification. Furthermore, Figure 4 also shows that a number of candidate genes (about 60%) identified by netSAM significantly overlap with known breast cancer genes in BCGS. Accordingly, we can conclude that netSAM is a more effective approach for identifying biomarkers.

Although BCGS consists of 452 genes based on the results of searching the related articles referenced in PubMed [31], until now, however, most of genes still have not been proved to be breast cancer susceptibility genes with absolute certainty. Thus, when these genes are used as true breast cancer genes to test the performance of our method, it would potentially cause some bias.

### 3.5. GO Analysis

Most cancers, including breast cancer, are complex disorders that are generally caused by multiple genes and their complex interactions. By mapping the 76 intersection genes identified by the netSAM to the gene ontology (GO) [7] terms, we found 11 GO functional categories, given in Table 1. The obtained GO terms are consistent to those in curated literature [32], which suggested that the above categories largely captures the functional facets of the breast cancer-specific gene network. Several cellular processes such as metabolism, cell proliferation and replication, apoptosis, inflammation, and cell cycle are known to be pivotal for tumorigenesis. The result of GO analysis indicates that our discovered signature has an enrichment score (ES) of 0.79, which means that identified oncogenes contain the majority of genes contributing to the enrichment score.

The full detail of Gene Ontology enrichment analysis is shown in Table 1. Tumor genes identified by netSAM are enriched in important biological processes catalogued in the Gene Ontology. From Table 1, it can be seen that the detected oncogenes are significantly enriched in GO terms of apoptosis, metabolism, immune response, and cell cycle. Inflammatory response is overrepresented and can be considered as potential candidate because chronic inflammation is widely believed to be a predisposing factor for cancer. These results suggested that the above categories largely captured the functional facets of the breast cancer specific gene.

### 3.6. KEGG Pathway Functional Analysis

Gene set enrichment analysis of Kyoto Encyclopedia of Genes and Genomes (KEGG) [8] pathway was conducted to find additional supporting evidence as described in Table 2. The enriched pathways were found. In the enriched pathways, TGF-beta, p53, and Notch and JAK-STAT signaling pathways are frequently reported to be related to breast tumor metastasis [33]. Notch signaling pathway may play essential role in the cross-talk between metastasis and relapse free. Recently, it has been found that *p53* activates the MAPK pathways through a feedback loop in human cancer. Moreover, we found that the detected genes were enriched for many known pathways, such as Apoptosis and Cell cycle. DAVID [34] genetic disease class category analysis indicated that the Benjamin *P* value of Apoptosis and Cell cycle is 1.1*E* − 6 and 3.3*E* − 4, respectively. Six hub genes (*TSPYL5, CD55, CCNE2, DCK, BBC3,* and *MUC1*) were all proved cancer-related hub genes. From Table 2, one can conclude that identified six genes significantly enriched in ECM, P53, and cell cycle pathway.

**Figure 5:**
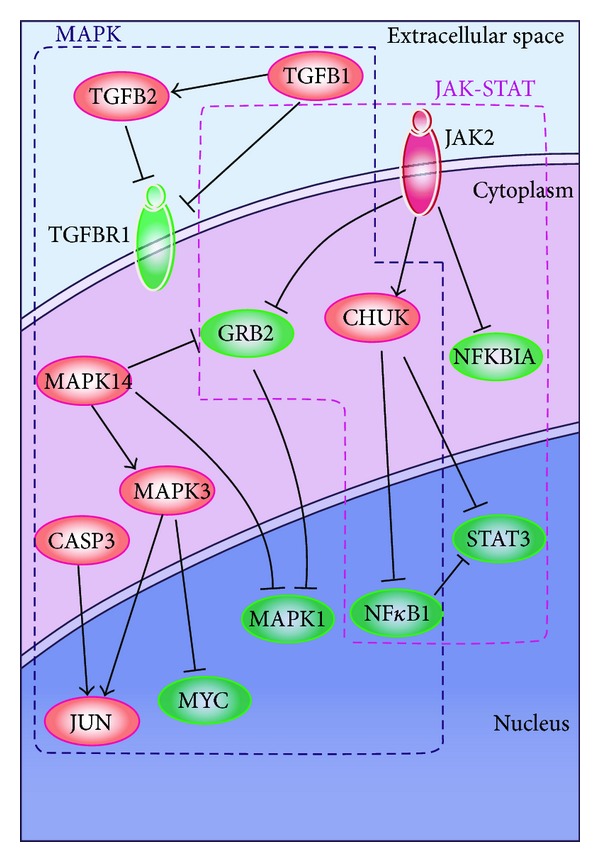
Signaling pathways highlighted in the identified cancer-related genetic network by netSAM on Wang et al. dataset, including MAPK and JAK-STAT pathways.

The signaling pathways depicted in Figure 5 include MAPK and JAK-STAT signaling pathways, which were highlighted in the top-ranked cancer-related genetic network identified by netSAM method from Wang et al. breast cancer dataset.

## 4. Conclusions

In this paper, we proposed netSAM to identify breast cancer-related genes from two benchmark breast cancer datasets (Wang et al. and Van De Vijver et al). Using netSAM, we identified six novel genes (*TSPYL5, CD55, CCNE2, DCK, BBC3,* and *MUC1*) as cancer biomarkers for predicting survival and metastasis in patients with breast cancer. Each of 6 genes in our signature not only has links to potential cancer relapse through the literature, they also have been shown in most cases to be directly linked to prognostic outcome, metastasis, and apoptosis. Furthermore, the six novel genes identified in our experiments are overlapped with the breast cancer gene set BCGS of literature curation. Further functional enrichment analysis and independent literature evidence also confirm that our identified potential cancer-causing genes are biologically reasonable, indicating the effectiveness of our method. Moreover, nearly 60% of the 119 oncogenes found by netSAM were certified as breast cancer susceptibility genes or known cancer-associated genes through literature mining. Our results indicate that the resulting signature possesses higher prediction precisions compared to previous work in the area and might be useful in predicting metastasis of breast cancer and facilitating treatment decisions. 

*TSPYL5* (TSPY-like 5), also known as *KIAA1750*, is involved in nucleosome assembly, a process which can alter the regulatory mechanisms of a cell [35], which is likely to occur in cancer. *TSPYL5* has been previously used as a prognostic biomarker in breast cancer [36]. In addition, it has been noted to play a role in the circulation of luteinizing hormone (LH), which is known to prompt tumor growth in breasts. Moreover, the individual gene (*TSPYL5*) is present in the 17 genes selected by Alexe et al. [3]. *CD55* has been used previously as a prognostic biomarker in gastric cancer. *CD55* has been shown to be important in breast cancer prognosis [37].

*CCNE2* encodes a protein similar to cyclin that serves as regulators of cyclin dependent kinase (CDK). A significant increase in the expression level of this gene was observed in tumor-derived cells. *CCNE2* has also been conformed to qualify as independent prognostic markers for lymph node-negative breast tumor patients and reported to have a predictive value in ER positive cases among breast cancer patients [9]. 

The *DCK* (deoxycytidine kinase) gene is required for the phosphorylation of several deoxyribonucleosides and their nucleoside analogs. It has been used to study resistance to chemotherapy in myeloid leukemia (AML) and breast cancer patients [21]. In addition, this particular gene may catalyze the metabolic activation of gemcitabine, a drug that has been used to treat several different types of cancer. However, the exact function of this gene is still unknown.

The *BBC3* gene, also known as *PUMA*, is located on human chromosome 19q13.3-q13.4 and is homologous with a *BCL2* family member. *BBC3* has a distinguished function in regulating other genes [38]. Many tumor genes are correlated with *BBC3*. The biological role for *BBC3* is to induce apoptosis via the mitochondrial apoptotic pathway. Furthermore, *BBC3* is also transcriptionally activated by the tumor suppressor *p53*, which is a key regulator of apoptosis and tumor genesis in breast cancer [22]. 

*MUC1* gene encodes a highly glycosylated protein located on the apical surface of mammary epithelia that is aberrantly overexpressed in approximately 90% of human breast cancers [39]. However, its role in cancer metastasis is yet less well understood. *MUC1* protein overexpression has been associated with cell adhesion inhibition as well as increased metastatic and invasive potential of tumor cells. This over-expression allows *MUC1* to interact with members of the *ERBB* family of receptor tyrosine kinases [40].

In the proposed netSAM procedure, a series of statistical methods and techniques were employed. Despite the difference in methodology, our analysis confirmed some of previous findings. For example, we also found the correlation of *ERBB2* and *MUC1* with breast cancer prognosis. Besides, when we applied traditional gene-based methods (*t*-test and lasso) to the gene expression datasets, we found that only a small part of the known tumor genes was identified as breast cancer-related genes. 

In conclusion, oncogenes found by netSAM can be used to stratify patients for treatment of the disease as well as extend perception to the disease mechanism for breast cancer, supply potential information in clinical decision-making, and help to reduce costs of therapy. However, these genes could not yet be fully justified with the current clinical knowledge, and further experimental validation is urgent. Differential genetic interaction networks have been proved very powerful for mapping the pathways that modulate/mediate essential cell functions. Our work demonstrated that a differential network-based inference method can provide a powerful tool for identifying associated genes in human disease.

Future work includes exploring other procedures for further improving accuracy and efficiency of detection, for example, using protein interaction network information. It is also believed that the incorporation of additional biological data and information would acquire better biomarkers for disease gene discovery.

## Figures and Tables

**Algorithm 1:**
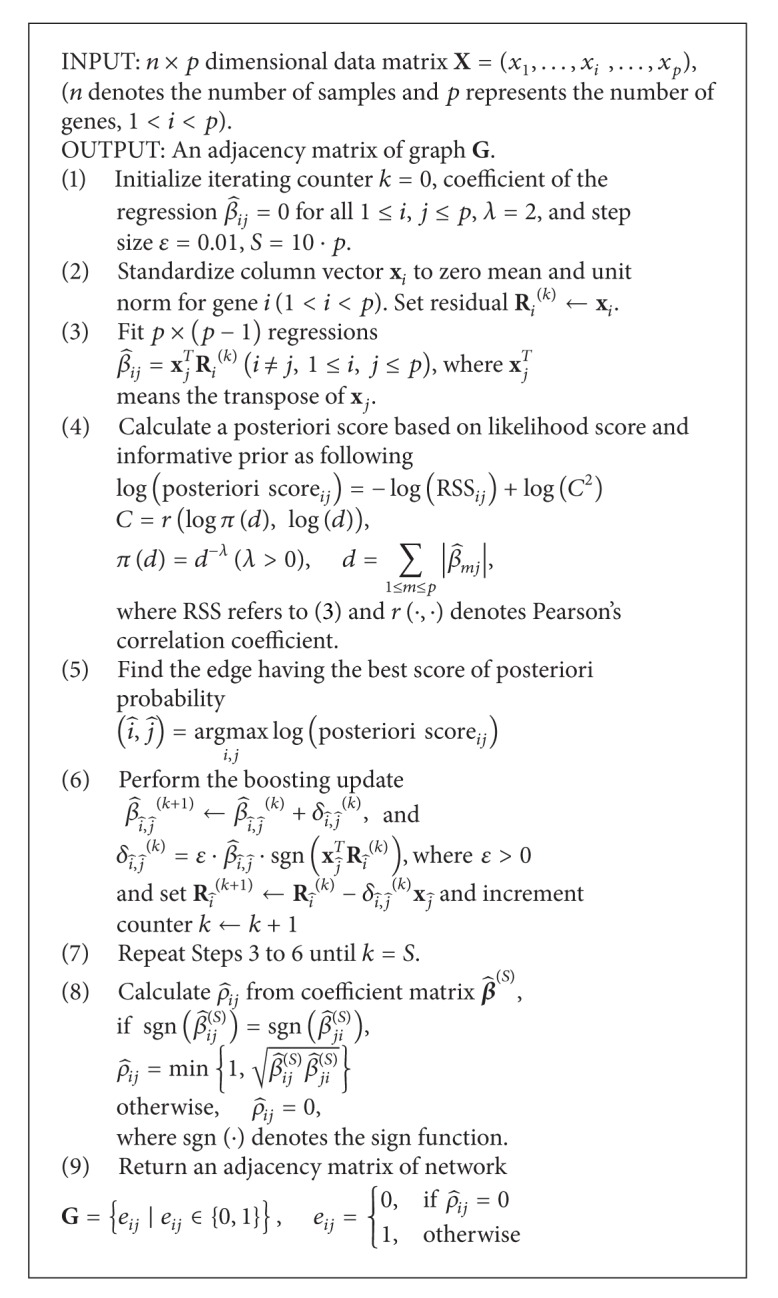
Posteriori score-based boosting regression algorithm for inferring networks as Step 2 of netSAM.

**Table 1:** Significantly enriched GO terms of biological process via BiNGO functional annotation analysis for the 76 intersection genes.

GO term	Hypergeometric test *P* value	Benjamini correction *P*-value	Frequency of mapped genes (%)	Fisher *P*-value
Immune system process	1.5280*E* − 14	1.7847*E* − 11	33.3	2.3*E* − 12
Cell cycle	3.5350*E* − 12	2.0645*E* − 9	20.4	1.3*E* − 12
Immune response	6.2486*E* − 12	2.4328*E* − 9	24.7	1.3*E* − 9
Cell division	1.5915*E* − 11	4.4740*E* − 9	18.2	1.3*E* − 11
Nuclear division	2.2983*E* − 11	4.4740*E* − 9	16.1	7.2*E* − 12
Apoptotic process	2.2983*E* − 11	4.4740*E* − 9	16.1	7.2*E* − 12
Metabolism	3.9513*E* − 11	5.7689*E* − 9	16.1	1.3*E* − 11
Cell proliferation	1.0537*E* − 10	1.2307*E* − 8	22.5	3.4*E* − 11
Inflammatory response	5.4845*E* − 8	4.2706*E* − 6	41.9	1.4*E* − 10
Response to stimulus	6.6080*E* − 5	1.9433*E* − 3	44.0	5.6*E* − 10
System development	5.1327*E* − 4	8.4436*E* − 3	31.1	2.3*E* − 11

**Table 2:** KEGG pathway functional analysis via DAVID for the 76 intersection genes.

KEGG pathway	Count	Frequency (%)	*P* value	Benjamin
Viral myocarditis	10	10.4	1.6*E* − 8	1.0*E* − 6
Apoptosis	8	8.3	3.3*E* − 8	1.1*E* − 6
Type I diabetes mellitus	8	8.3	1.0*E* − 7	1.7*E* − 6
Autoimmune thyroid disease	8	8.3	4.2*E* − 7	5.3*E* − 6
Cell cycle	9	9.4	3.1*E* − 5	3.3*E* − 4
TGF-beta signaling pathway	8	8.3	1.7*E* − 4	1.2*E* − 3
Notch signaling pathway	6	6.2	3.9*E* − 3	2.4*E* − 2
ECM-receptor interaction	5	5.2	8.3*E* − 3	4.8*E* − 2
JAK-STAT signaling pathway	7	7.3	1.2*E* − 2	6.2*E* − 2
P53 signaling pathway	4	4.2	4.9*E* − 2	2.1*E* − 1
Immune network	3	3.1	8.0*E* − 2	3.0*E* − 1

## Appendix

See, Algorithm 1.

## References

[1] Van De Vijver MJ, He YD, van 't Veer LJ (2002). A gene-expression signature as a predictor of survival in breast cancer. *New England Journal of Medicine*.

[2] Sotiriou C, Neo S-Y, McShane LM (2003). Breast cancer classification and prognosis based on gene expression profiles from a population-based study. *Proceedings of the National Academy of Sciences of the United States of America*.

[3] Alexe G, Alexe S, Axelrod DE (2006). Breast cancer prognosis by combinatorial analysis of gene expression data. *Breast Cancer Research*.

[4] Sotiriou C, Piccart MJ (2007). Taking gene-expression profiling to the clinic: when will molecular signatures become relevant to patient care?. *Nature Reviews Cancer*.

[5] Desmedt C, Piette F, Loi S (2007). Strong time dependence of the 76-gene prognostic signature for node-negative breast cancer patients in the TRANSBIG multicenter independent validation series. *Clinical Cancer Research*.

[6] Subramanian A, Kuehn H, Gould J, Tamayo P, Mesirov JP (2007). GSEA-P: a desktop application for gene set enrichment analysis. *Bioinformatics*.

[7] Ashburner M, Ball CA, Blake JA (2000). Gene ontology: tool for the unification of biology. *Nature Genetics*.

[8] Ogata H, Goto S, Sato K, Fujibuchi W, Bono H, Kanehisa M (1999). KEGG: Kyoto encyclopedia of genes and genomes. *Nucleic Acids Research*.

[9] Sotiriou C, Pusztai L (2009). Gene-expression signatures in breast cancer. *New England Journal of Medicine*.

[10] Wu K, House L, Liu W, Cho WCS (2012). Personalized targeted therapy for lung cancer. *International Journal of Molecular Sciences*.

[11] Chuang H-Y, Lee E, Liu Y-T, Lee D, Ideker T (2007). Network-based classification of breast cancer metastasis. *Molecular Systems Biology*.

[12] Wu X, Jiang R, Zhang MQ, Li S (2008). Network-based global inference of human disease genes. *Molecular Systems Biology*.

[13] Fröhlich H (2011). Network based consensus gene signatures for biomarker discovery in breast cancer. *PloS ONE*.

[14] Chen L, Xuan J, Riggins RB, Clarke R, Wang Y (2011). Identifying cancer biomarkers by network-constrained support vector machines. *BMC Systems Biology*.

[15] Ideker T, Krogan NJ (2012). Differential network biology. *Molecular Systems Biology*.

[16] Valcárcel B, Würtz P, Seich al Basatena NK (2011). A differential network approach to exploring differences between biological states: an application to prediabetes. *PLoS ONE*.

[17] Gambardella G, Moretti MN, de Cegli R (2013). Differential network analysis for the identification of condition-specific pathway activity and regulation. *Bioinformatics*.

[18] Iancu OD, Oberbeck D, Darakjian P (2013). Differential network analysis reveals genetic effects on catalepsy modules. *PLoS ONE*.

[19] West J, Bianconi G, Severini S, Teschendorff AE (2012). Differential network entropy reveals cancer system hallmarks. *Scientific Reports*.

[20] Shen CY, Huang Y, Liu Y (2011). A modulated empirical bayes model for identifying topological and temporal estrogen receptor alpha regulatory networks in breast cancer. *BMC Systems Biology*.

[21] Costantino CL, Witkiewicz AK, Kuwano Y (2009). The role of HuR in gemcitabine efficacy in pancreatic cancer: HuR up-regulates the expression of the gemcitabine metabolizing enzyme deoxycytidine kinase. *Cancer Research*.

[22] Cotter TG (2009). Apoptosis and cancer: the genesis of a research field. *Nature Reviews Cancer*.

[23] Morrison JL, Breitling R, Higham DJ, Gilbert DR (2005). GeneRank: using search engine technology for the analysis of microarray experiments. *BMC Bioinformatics*.

[24] Smoot ME, Ono K, Ruscheinski J, Wang P-L, Ideker T (2011). Cytoscape 2.8: new features for data integration and network visualization. *Bioinformatics*.

[25] Skog J, Würdinger T, van Rijn S (2008). Glioblastoma microvesicles transport RNA and proteins that promote tumour growth and provide diagnostic biomarkers. *Nature Cell Biology*.

[26] Tibshirani R (2011). Regression shrinkage and selection via the lasso: a retrospective. *Journal of the Royal Statistical Society B*.

[27] Geeven G, van Kesteren RE, Smit AB, de Gunst MCM (2012). Identification of context-specific gene regulatory networks with GEMULA—gene expression modeling using LAsso. *Bioinformatics*.

[28] Van den Bulcke T, Van Leemput K, Naudts B (2006). SynTReN: a generator of synthetic gene expression data for design and analysis of structure learning algorithms. *BMC Bioinformatics*.

[29] Wang Y, Klijn JGM, Zhang Y (2005). Gene-expression profiles to predict distant metastasis of lymph-node-negative primary breast cancer. *Lancet*.

[30] Edgar R, Domrachev M, Lash AE (2002). Gene Expression Omnibus: NCBI gene expression and hybridization array data repository. *Nucleic Acids Research*.

[31] McEntyre J, Lipman D (2001). PubMed: bridging the information gap. *Canadian Medical Association Journal*.

[32] Lv W, Yang T (2012). Identification of possible biomarkers for breast cancer from free fatty acid profiles determined by GC-MS and multivariate statistical analysis. *Clinical Biochemistry*.

[33] Vogelstein B, Kinzler KW (2004). Cancer genes and the pathways they control. *Nature Medicine*.

[34] Dennis G, Sherman BT, Hosack DA (2003). DAVID: database for annotation, visualization, and integrated discovery. *Genome Biology*.

[35] Henikoff S (2008). Nucleosome destabilization in the epigenetic regulation of gene expression. *Nature Reviews Genetics*.

[36] Sieuwerts AM, Look MP, Meijer-Van Gelder ME (2006). Which cyclin E prevails as prognostic marker for breast cancer? Results from a retrospective study involving 635 lymph node-negative breast cancer patients. *Clinical Cancer Research*.

[37] Madjd Z, Durrant LG, Bradley R, Spendlove I, Ellis IO, Pinder SE (2004). Loss of *CD55* is associated with aggressive breast tumors. *Clinical Cancer Research*.

[38] Raj L, Ide T, Gurkar AU (2011). Selective killing of cancer cells by a small molecule targeting the stress response to ros. *Nature*.

[39] Ibrahim NK, Yariz KO, Bondarenko I (2011). Randomized phase II trial of letrozole plus Anti-MUC1 antibody AS1402 in hormone receptor-positive locally advanced or metastatic breast cancer. *Clinical Cancer Research*.

[40] Kufe DW (2012). Muc1-c oncoprotein as a target in breast cancer: activation of signaling pathways and therapeutic approaches. *Oncogene*.

